# A clinician-based comparative study of large language models in answering medical questions: the case of asthma

**DOI:** 10.3389/fped.2025.1461026

**Published:** 2025-04-25

**Authors:** Yong Yin, Mei Zeng, Hansong Wang, Haibo Yang, Caijing Zhou, Feng Jiang, Shufan Wu, Tingyue Huang, Shuahua Yuan, Jilei Lin, Mingyu Tang, Jiande Chen, Bin Dong, Jiajun Yuan, Dan Xie

**Affiliations:** ^1^ Department of Respiratory Medicine, Hainan Branch, Shanghai Children's Medical Center, School of Medicine, Shanghai Jiao Tong University, Sanya, China; ^2^ Department of Respiratory Medicine, Shanghai Children’s Medical Center, Shanghai Jiao Tong University School of Medicine, Shanghai, China; ^3^ Pediatric AI Clinical Application and Research Center, Shanghai Children’s Medical Center, Shanghai, China; ^4^ Shanghai Engineering Research Center of Intelligence Pediatrics (SERCIP), Shanghai, China; ^5^ Department of Performance, Shanghai Children’s Medical Center, Shanghai Jiao Tong University School of Medicine, Shanghai, China; ^6^ Department of Discipline Inspection and Supervision, Shanghai Children’s Medical Center, Shanghai Jiao Tong University School of Medicine, Shanghai, China; ^7^ Medical Department of Shanghai Children’s Medical Center, Shanghai Jiao Tong University School of Medicine, Shanghai, China

**Keywords:** large language models (LLMs), pediatric asthma, medical question answering, clinical decision support, AI in healthcare

## Abstract

**Objective:**

This study aims to evaluate and compare the performance of four major large language models (GPT-3.5, GPT-4.0, YouChat, and Perplexity) in answering 32 common asthma-related questions.

**Materials and methods:**

Seventy-five clinicians from various tertiary hospitals participated in this study. Each clinician was tasked with evaluating the responses generated by the four large language models (LLMs) to 32 common clinical questions related to pediatric asthma. Based on predefined criteria, participants subjectively assessed the accuracy, correctness, completeness, and practicality of the LLMs' answers. The participants provided precise scores to determine the performance of each language model in answering pediatric asthma-related questions.

**Results:**

GPT-4.0 performed the best across all dimensions, while YouChat performed the worst in all dimensions. Both GPT-3.5 and GPT-4.0 outperformed the other two models, but there was no significant difference in performance between GPT-3.5 and GPT-4.0 or between YouChat and Perplexity.

**Conclusion:**

GPT and other large language models can answer medical questions with a certain degree of completeness and accuracy. However, clinical physicians should critically assess internet information, distinguishing between true and false data, and should not blindly accept the outputs of these models. With advancements in key technologies, LLMs may one day become a safe option for doctors seeking information.

## 1 Introduction

Asthma is a major chronic respiratory disease worldwide, affecting the health and quality of life of millions of people. In a multinational, multicenter study involving 453,473 subjects, it was found that 6.3% of children, 7.9% of adolescents, and 3.4% of adults were diagnosed with asthma by a doctor. Moreover, in middle-to-low-income countries, many individuals with severe asthma symptoms were not using inhaled corticosteroids (1). In China, 15.5% of asthma patients reported at least one emergency room visit, and 7.2% of patients reported at least one hospitalization due to worsening respiratory symptoms (2). Despite receiving high-intensity treatment, most children with poorly controlled symptoms can achieve improved asthma control when they adhere to the basic principles of asthma management (3). Frequent and severe asthma attacks can be fatal, and effective asthma management and treatment require close cooperation between patients, doctors, and caregivers. Therefore, improving the provision of accurate health information and personalized counseling is crucial for the self-management of asthma patients.

A survey of online health behaviors of Americans revealed that more than one-third of Americans turn to the Internet to diagnose health problems (4). Large Language Models (LLMs), such as GPT, are AI tools designed to process and generate text. They have been widely applied to various tasks and have demonstrated excellent performance in the medical field (5). LLMs will increasingly be used for information retrieval, automated summarization of literature notes, answering medical questions, and even as interactive tools in medical education (6, 7). This not only helps patients access important disease-related information more quickly but also supports the decision-making process of healthcare professional (8). However, Information errors, privacy issues, and ethical challenges and potential harm to patient care remain significant challenges (9). Ethical issues, including data privacy and breaches, must be addressed. In both medical and non-medical education, students are vulnerable to misinformation, hindering the development of critical thinking skills. The lack of mechanisms to ensure the accuracy of LLM outputs limits their use in clinical settings, where misinformation can have fatal consequences (7).

In this study, we aim to evaluate and compare the performance of four selected Large Language Models (GPT-3.5, GPT-4.0, YouChat, and Perplexity) in answering clinical questions related to pediatric asthma. The evaluation includes four dimensions: accuracy, precision, completeness, and practicality, combined with insights from professionals for a comprehensive assessment. Our findings may provide valuable insights into the clinical application of LLMs as medical auxiliary tools and promote clinical decision-making.

## 2 Article type

This study is an Original Research Article that evaluates and compares the performance of four major large language models (GPT-3.5, GPT-4.0, YouChat, and Perplexity) in answering 32 common asthma-related questions.

## 3 Material and methods

### 3.1 Model selection

Based on previous research, user volume, and training methodologies, this study selected four models for investigation: ChatGPT 3.5, ChatGPT 4.0, YouChat, and Perplexity. ChatGPT 3.5 and ChatGPT 4.0 were trained on predefined datasets and did not connect to the internet after their launch. ChatGPT 4.0 utilizes a more extensive and diverse pre-training dataset compared to ChatGPT 3.5, along with advanced training techniques such as more effective model optimization algorithms and smarter parameter initialization methods. The version of YouChat used in this study is the basic version, which extends ChatGPT 3.5 by integrating an internet search function. Similarly, the Perplexity version used is the basic one, functioning as an AI-powered search engine that combines proprietary language models with real-time web retrieval to generate responses.

### 3.2 Question selection and answering with large language models

The equations should be inserted in editable format from the equation editor. We selected 32 common asthma-related questions from the article “One hundred key issues on Chinese Children's Asthma Action Plan” published in the Chinese Journal of Practical Pediatrics to test the model (10). On the one hand, these questions were selected after consultation with three pediatric respiratory asthma experts and reflected the main aspects of asthma management, such as diagnosis, treatment, prevention and follow-up. On the other hand, the selection process was designed to cover essential topics related to the concerns of clinicians and patients and their families in the clinical setting. All questions were posed and recorded in Chinese, and we translated them into English for presentation (see Table 1). The prompt for all models was set as: “Assume you are an expert in the field of pediatrics, and the following questions are all related to pediatrics. Please answer the following questions in less than 500 words.” The questions were inputted in the exact same order and content for all models. To ensure consistency and eliminate potential influence on clinician ratings, we manually removed all hyperlinks, quotation marks, and web-related formatting from all model responses. All answers were presented in a uniform plain text format and model identities were anonymized. This standardization ensured that assessments were based solely on the accuracy, correctness, completeness, and utility of the content, and not on the presence or absence of supporting links or reference formats. To evaluate the internal stability of the models, we created five dialogues using the same input method. The project team members jointly assessed the stability of the five responses, and the results were recorded on a ten-point scale, with a minimum of 1 and a maximum of 10.

**Table 1:** Questions used to test the performance of LLMs.

32 Questions Related to Childhood Asthma	
Question 1	What is asthma?
Question 2	Is asthma hereditary?
Question 3	What are the differences and similarities between asthma in children and adult asthma?
Question 4	What are the clinical features of asthma in children?
Question 5	How is bronchial asthma in children diagnosed?
Question 6	Can recurrent wheezing in infancy develop into asthma?
Question 7	What are the comorbid conditions of asthma?
Question 8	What impact does allergic rhinitis (AR) have on asthma?
Question 9	What are the common tests for childhood asthma?
Question 10	Can childhood asthma be cured?
Question 11	Does long-term ICS treatment affect the growth and development of children?
Question 12	Which children with asthma are eligible for allergen specific immune therapy (AIT)?
Question 13	Which children with asthma are eligible for biological treatments such as monoclonal antibodies?
Question 14	Why is it important to manage asthma in children?
Question 15	Why should children with asthma have regular follow-up visits to the hospital? How often should these visits occur?
Question 16	What are the main components of follow-up visits for children with asthma?
Question 17	What are the early preventive measures for asthma?
Question 18	What are common allergens? Why do children with asthma need allergen testing?
Question 19	What are dust mites? How can dust mite allergies be prevented?
Question 20	Which pet dander is likely to cause allergies?
Question 21	How can pollen allergies be managed?
Question 22	Can children with asthma receive vaccinations?
Question 23	What is the relationship between asthma attacks and upper respiratory infections?
Question 24	Can children with asthma exercise? How should they exercise?
Question 25	Can exercise induce asthma attacks? How can exercise-induced asthma attacks be prevented?
Question 26	What climate changes are likely to trigger asthma attacks? How can these be prevented?
Question 27	What are the adverse effects of cigarette smoke exposure on children with asthma? How can this be prevented?
Question 28	What factors are likely to cause acute asthma attacks during outdoor activities or travel?
Question 29	What signs can predict an acute attack of asthma in children?
Question 30	How can the severity of an acute asthma attack in children be assessed?
Question 31	How can severe acute asthma attacks be prevented?
Question 32	What emergency medications should be readily available at home or nearby for children with asthma?

### 3.3 Model evaluation dimensions

This study designed the questionnaire from the perspective of doctors. The questionnaire evaluates the responses of different models based on four dimensions: “accuracy,” “correctness,” “completeness,” and “practicality.” “Accuracy” is defined as the degree to which the model's answer is relevant to the question, reflecting the model's ability to understand the user's query. “Correctness” refers to the extent to which the model's answer aligns with the clinical experience and guidelines of the respondents. “Completeness” is defined as the thoroughness of the model's answer compared to clinical experience and guidelines. “Practicality” refers to the extent to which the model's answer is applicable in daily clinical practice, reflecting the model's ability to solve real-world problems. The results are recorded on a ten-point scale, with “unable to answer” responses scored as 0 and other answers scored between 1 and 10. The definitions of the four evaluation dimensions are placed on the first page of the questionnaire to clearly inform the respondents and facilitate accurate evaluation.

### 3.4 Questionnaire design

Each questionnaire contained thirty-two questions, arranged in the same order, with answers generated by different large language models. Participants were instructed to provide clear and unambiguous answers based on existing clinical guidelines. The four model-generated answers for each question were presented in random order, and participants were not informed which model corresponded to each answer. To improve the quality of questionnaire completion, we set a time limit for answering the questions. The questionnaires were then distributed in paper form to 75 clinicians and collected uniformly. This study was conducted from January to May 2024.

### 3.5 Participant inclusion

The evaluators in this study met the following criteria: (1) Hold a Master's degree in medicine or higher; (2) be under 60 years of age; (3) Have worked in the pediatric department of a tertiary hospital.

### 3.6 Questionnaire quality control

We implemented quality control for the questionnaires based on the following criteria: (1) Assigning a high score to responses with obvious errors/deficiencies was considered one quality control anomaly; (2) Completing the questionnaire in less than 2 h was counted as one quality control anomaly; (3) Having three responses with clearly outlier scores was counted as one quality control anomaly. If there were fewer than three such scores, it was counted as three instances. A sample was deemed to have failed quality control if it exhibited five instances of quality control anomalies. Only samples that passed quality control were included in the analysis.

### 3.7 Inter-rater reliability analysis

To assess the consistency of raters in rating different models, we conducted an inter-rater reliability analysis using the Intraclass Correlation Coefficient (ICC). The ICC is a widely used metric to measure the level of agreement between raters when rating continuous data. In this study, ICC values were calculated for four rating aspects—accuracy, completeness, correctness and practicality—using different models. Higher ICC values indicate better agreement between raters. The final results are shown in Supplementary Figure S1, where it can be observed that Perplexity and YouChat provided the most consistent ratings, with ICC values ranging from 0.85–0.91 across all aspects, indicating a high level of inter-rater agreement. In contrast, GPT-4.0 showed the greatest variation in raters' scores, particularly for Correctness and Practicality.

### 3.8 Statistical analysis

All data analysis was conducted using R 4.3.3. To comprehensively understand the responses of the four major language models to asthma-related clinical questions, we calculated the average score for each question answered by each model and presented the results through bar charts. Next, we calculated the average score for each model across all evaluative dimensions per question to examine the distinct responses provided by each model. Sankey diagrams were used to describe the commonalities and differences in cumulative scores for the top five and bottom five questions among the four models. To assess differences between the models, we first determined the average score for each question across different models and then performed hypothesis testing using Tukey's *post hoc* test. We then used Tukey's *post hoc* test to compare the performance of the four models across various dimensions. Finally, we utilized Tukey's *post hoc* test to evaluate the significance of differences within each model across different dimensions.

## 4 Results

### 4.1 Questionnaire distribution and recall

The research distributed a total of 75 questionnaires, all of which were returned and passed quality control, yielding a qualification rate of 100%.

### 4.2 Evaluation of LLMs' performance

**Figure 1:**
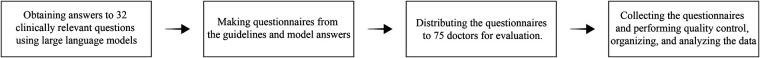
Flowchart.

**Figure 2:**
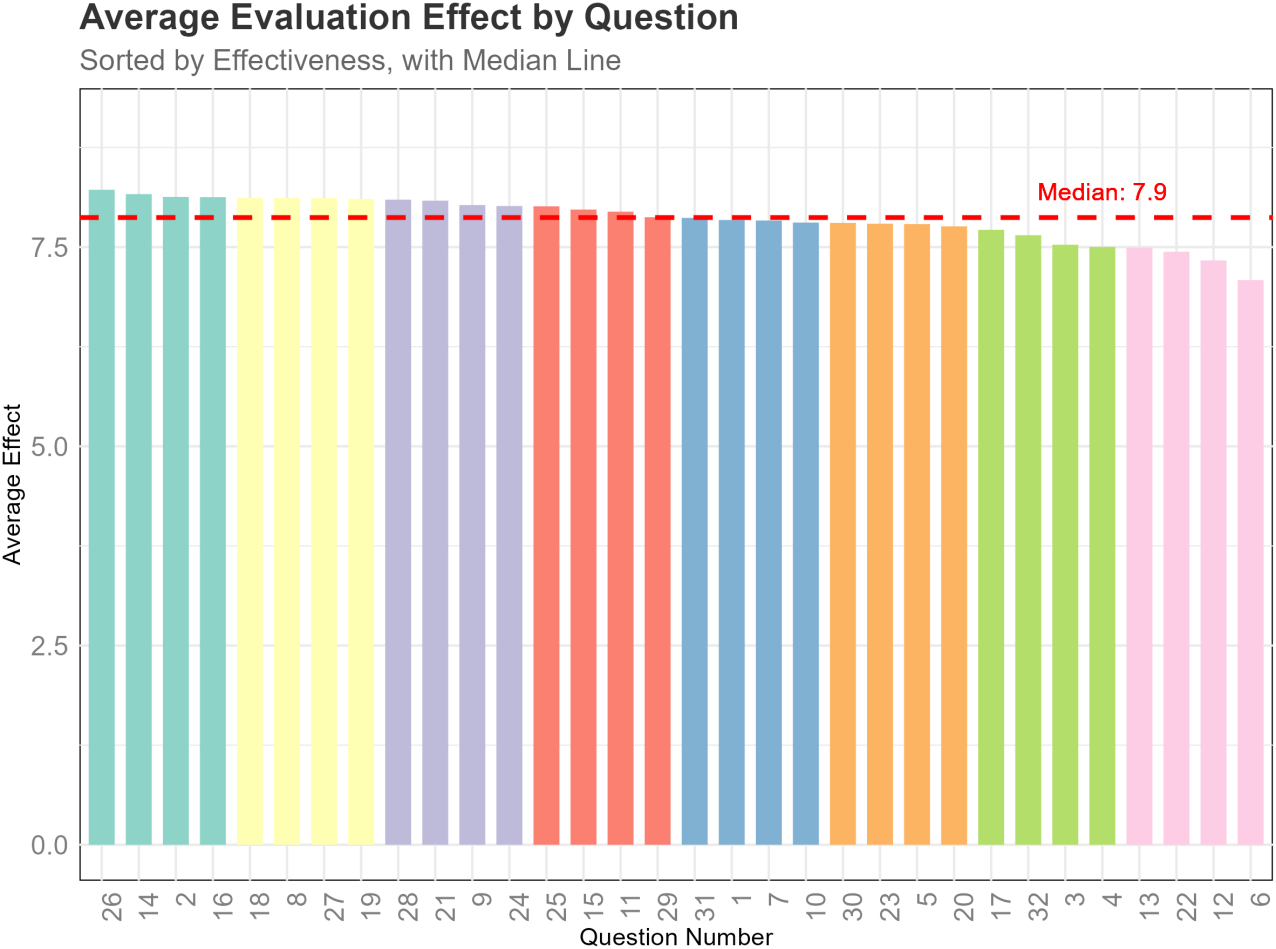
The average score of each question for all models.

Table 1 lists all the questions included in the 32 questionnaires. Figure 1 shows the flowchart of the study. Figure 2 shows the responses of the large language models (LLMs) to all questions. In the questionnaires, the median score for all questions answered by the LLMs was 7.9, with the highest scores for questions 26, 14, 2, 16, and 18, and the lowest scores for questions 6, 12, 22, 13, and 4. This indicates that the LLMs performed excellently in addressing the genetic causes, management strategies, and prevention of childhood asthma, but showed some weaknesses in addressing the clinical characteristics, early diagnosis, and specific treatments (such as allergen-specific immunotherapy and monoclonal antibody treatments) for childhood asthma.

**Figure 3:**
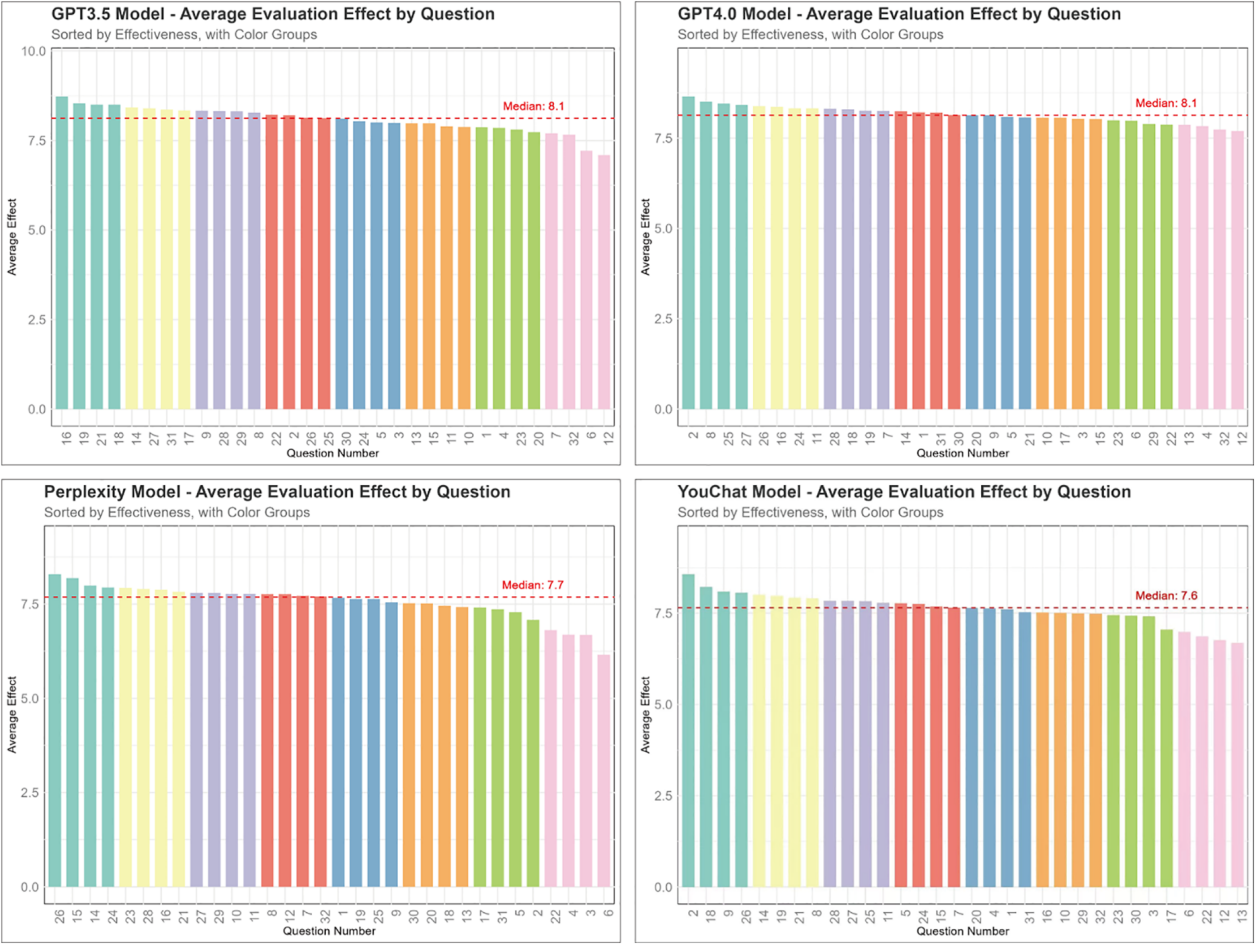
The average score of each question for different models.

Figure 3 displays the scores of different models on each question. ChatGPT 3.5 and ChatGPT 4.0 had higher median scores, both at 8.1, while Perplexity and YouChat had lower median scores, at 7.7 and 7.6, respectively.

**Figure 4:**
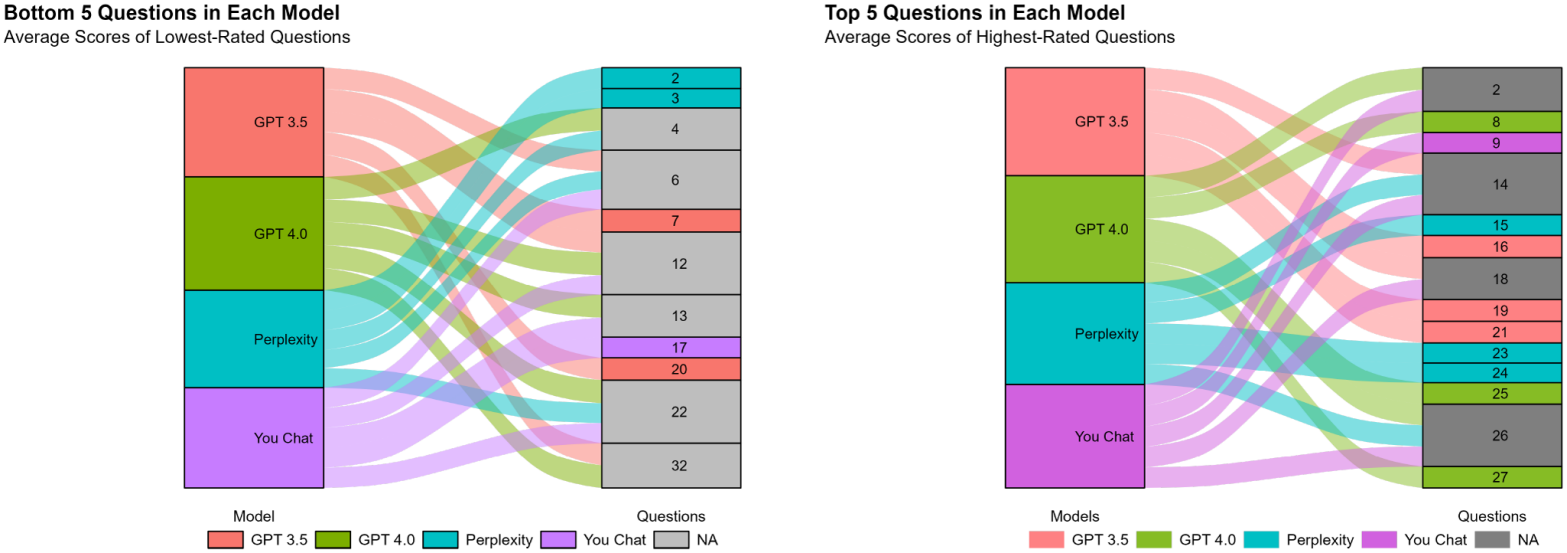
Sankey diagram of the questionnaire.

Figure 4 illustrates the differences and similarities between the top five and bottom five questions answered by the various models. Our findings indicate that multiple models demonstrated proficiency in answering questions 2, 14, 18, and 26, suggesting that LLMs are more adept at addressing questions related to genetic causes, management measures, and the prevention of childhood asthma. The GPT 4.0 demonstrated particular proficiency in responding to the questions with the highest scores. However, in the case of the questions with the lowest scores, multiple models exhibited less impressive performance on questions 6, 12, 22, and 32. This indicates that the LLMs (even with GPT 4.0) were less adept at answering questions pertaining to early identification and prevention of childhood asthma, personalized treatment, prevention, and emergency care management.

### 4.3 Comparison in different dimensions of each model

**Figure 5:**
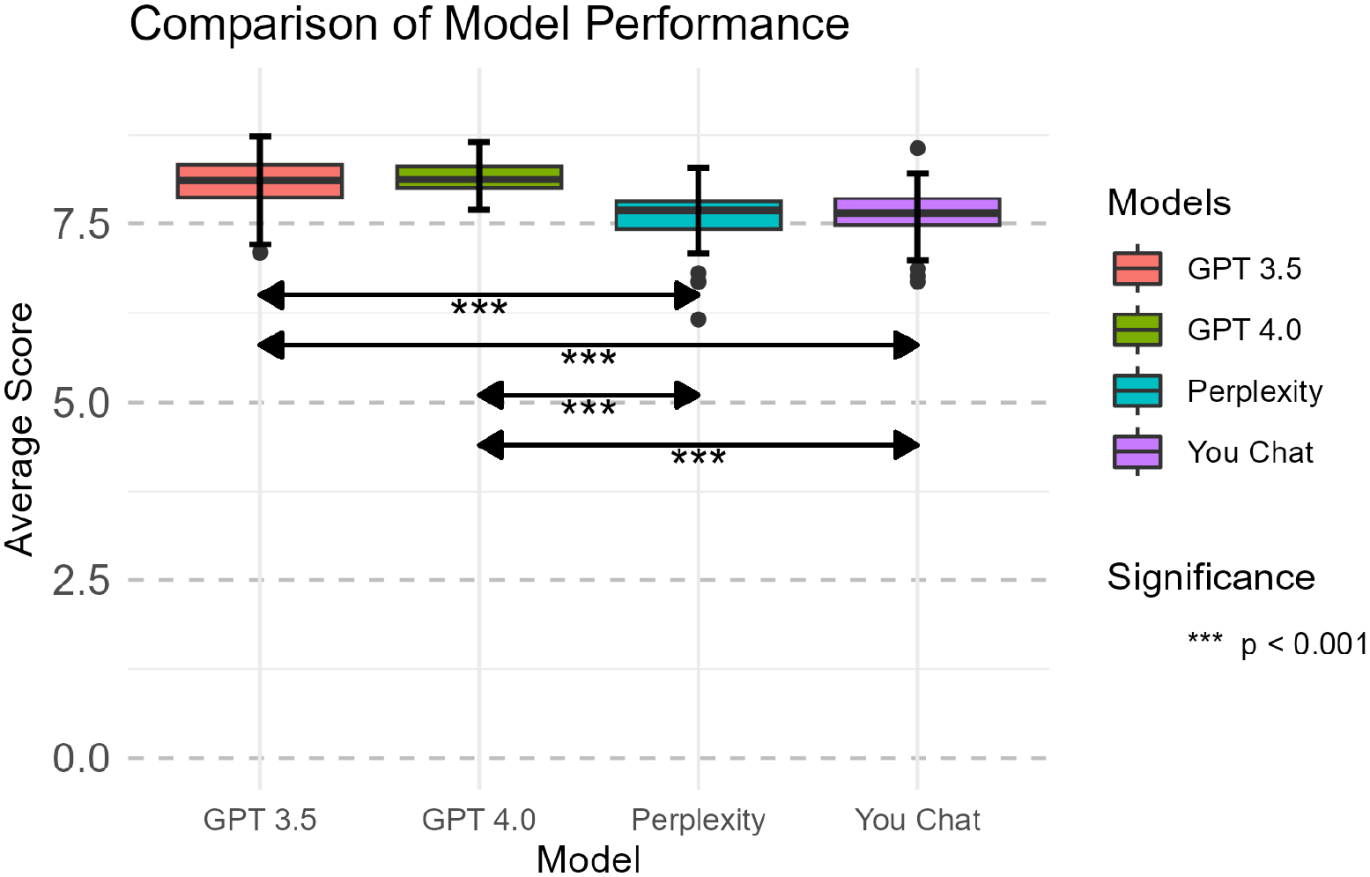
Comparison of average scores across all dimensions between models.

Figure 5 illustrates the average scores of different models across all questions. GPT 3.5 and GPT 4.0 significantly outperformed Perplexity and You Chat, exhibiting more stable and higher scores. There was no significant difference in performance between GPT 3.5 and GPT 4.0, with their median scores being nearly identical. Similarly, there was no significant difference between Perplexity and You Chat, with their median scores being close to each other.

**Figure 6:**
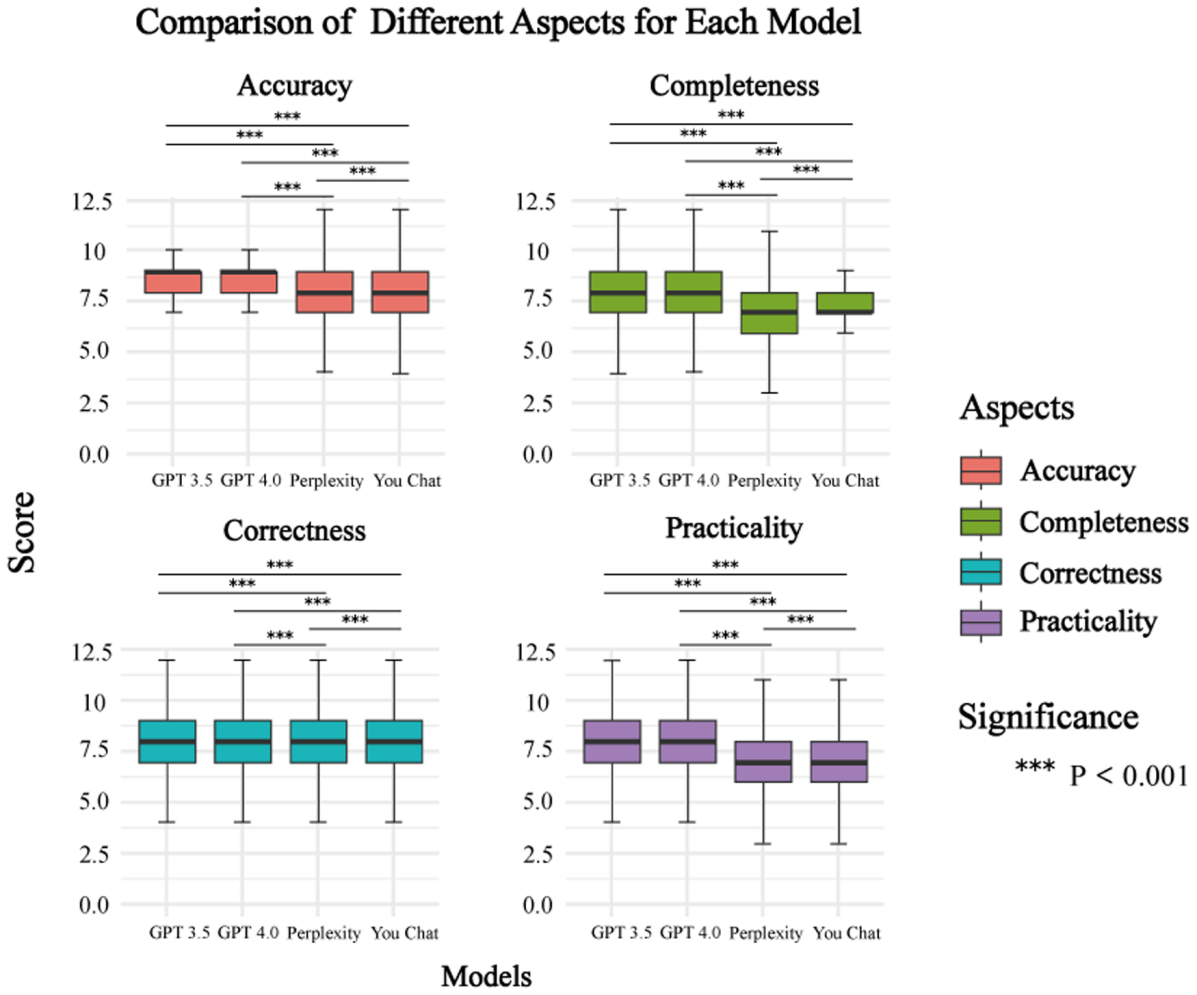
Comparison of average scores across different dimensions between models.

Figure 6 shows that the GPT-4.0 performed better on all four assessment dimensions, although statistical analyses showed no significant difference between the GPT-4.0 and GPT-3.5. Conversely, YouChat had the lowest performance in all aspects, putting it at a disadvantage compared to the other three models.

## 5 Discussions

Artificial intelligence is increasingly being applied in various medical projects, including radiological image analysis (11), aiding diagnosis in complex cases (12), personalized treatment (13), anesthesia depth monitoring and control (14), and drug development and utilization (15). A study evaluated ChatGPT's performance on the United States Medical Licensing Examination (USMLE), and the results showed that ChatGPT met or nearly met the passing threshold without any specialized training or reinforcement (16). The LLM demonstrated strong performance in making final diagnoses across 36 clinical cases, achieving an accuracy rate of 76.9% (17). Importantly, compared to other decision support tools, LLMs not only incorporate more patient-specific information to generate more targeted recommendations but also encourage brainstorming, prompting doctors to consider diagnoses and treatments they might otherwise overlook. These results suggest that large language models may have the potential to aid in medical education and assist in clinical decision-making.

In this study, all the major language models performed well in answering a range of clinically relevant questions, with particular excellence in the areas of asthma causes, treatment and prevention. This is probably because these topics are of greater public interest and there are more sources of information available, resulting in more training data and consequently higher scores. For asthma diagnosis and new treatments, the LLMs showed less stable performance, indicating a need for more recent data training in these areas.

GPT and other large language models can answer medical questions with a certain degree of completeness and accuracy. Our results indicate that while GPT-4.0 demonstrated the highest scores across all dimensions, the statistical analysis revealed no significant difference between GPT-4.0 and GPT-3.5. This suggests that both models perform comparably in medical question answering, and the choice between them may depend on factors beyond numerical scores. Despite this, we still recommend GPT-4.0 due to its qualitative advantages over GPT-3.5, including a larger database, more advanced training data, improved model architecture, and better integration with clinical guidelines. These factors enable GPT-4.0 to understand and generate more accurate and effective information. Additionally, qualitative feedback from clinicians suggests that GPT-4.0 provides smoother and more contextually relevant responses, making it more reliable in real-world medical scenarios. In the top five questions (Question 5: Is asthma hereditary? Question 8: What is the impact of allergic rhinitis (AR) on asthma? Question 25: Can exercise induce asthma attacks? How to prevent exercise-induced asthma attacks? Question 26: What climate changes can trigger asthma attacks? How to prevent them? Question 27: What adverse effects does cigarette exposure have on children with asthma? How to prevent them?), GPT-4.0 did an excellent job of answering questions about asthma heredity, triggers, and preventive measures. However, GPT-4.0 showed weaker capabilities in handling questions related to asthma management (vaccination) and treatment strategies (including emergency, immunotherapy, or biologic treatments). For some new asthma treatments, such as desensitization therapy and monoclonal antibody therapies, future model training should emphasize updating the database in these areas. If LLMs could be trained by reliable experts, it could rapidly improve and transform the dissemination of medical knowledge. Providing more and more disease information through LLMs could help address the growing prevalence of asthma.

Although YouChat performed the worst of all models, it significantly outperformed the other three models in answering questions about accurately diagnosing asthma (Question 9: What are common tests for childhood asthma?) and identifying and managing allergens (Question 18: What are common allergens? Why do children with asthma need allergen testing?). These interventions are complementary and form a systematic approach to comprehensive asthma management, demonstrating that each model has strengths in different aspects of disease management.

However, there are several limitations to this study. First, the sample size is relatively small (75 doctors), which may affect the generalizability of the results. Second, there may be biases in the questionnaire design, as the selection and phrasing of questions could influence the models' responses. Additionally, this study focuses solely on pediatric asthma questions, different medical domains might yield different results. Future research could expand the sample size and diversity of questions to improve the generalizability and reliability of the findings. It may also consider evaluating the models' performance in various medical fields (e.g., hypertension, diabetes) to gain a comprehensive understanding of their potential applications in medicine. Furthermore, research could explore ways to further enhance the training data and model architecture to improve their performance in specialized fields. Although the models performed well in this study, in practice, LLMs may give incorrect responses when faced with prompts that do not have a single correct answer, and if they present these responses in a convincing manner, users might believe their accuracy (18). Therefore, in practical use, doctors should use LLMs as supplementary and enhanced support rather than relying solely on their responses (19).

While our findings suggest that large language models (LLMs) such as GPT-4.0 have great potential as tools for clinical decision support, it is important to recognize the ethical risks and challenges they pose—particularly the risk of misinformation. For example, if an LLM suggests the use of an outdated or contraindicated asthma medication without considering the clinical context, this could lead to harmful outcomes-especially if the recommendation is followed without expert review. From an ethical perspective, the use of LLMs also raises questions about responsibility and accountability. Unlike human clinicians, LLMs do not have intent, awareness, or professional responsibility, making it difficult to determine who is liable if AI-generated content causes harm. In addition, LLMs responses may reflect biases in their training data or generate information that sounds accurate but not to. To mitigate these risks, several strategies should be implemented: (1) Human oversight: All LLM-generated content should be reviewed by qualified healthcare professionals before being used in clinical practice. (2) Transparency and interpretability: Developers should improve how LLMs explain their answers and ensure that the system can flag low-confidence or uncertain answers. (3) User training: Clinicians and other users should be trained to understand the limitations of LLMs and to use their results critically. (4) Ongoing monitoring: The performance of LLMs should be regularly reviewed in real-world settings to ensure continued safety and accuracy.

Based on the above, doctors still need to receive proper education and continuously update their knowledge through various traditional evidence-based educational methods. It is crucial to apply critical thinking to the information provided by LLMs and regard it as a supplement to their clinical knowledge and experience. Otherwise, clinicians can be easily misled. Currently, whether in terms of data or training, large language models do not seem capable of replacing the unique intellectual abilities of humans. Clinicians need to be very vigilant and apply all evaluative and critical measures to the information provided before establishing such tools as support for clinical decision-making. In the future, with advancements in key technologies and the resolution of diagnostic blind spots and data privacy issues, large language models have the potential to become important tools for improving human healthcare.

## 6 Conclusion

GPT and other large language models can answer medical questions with a certain degree of completeness and accuracy. However, clinical physicians should critically assess internet information, distinguishing between true and false data, and should not blindly accept the outputs of these models. With advancements in key technologies, LLMs may one day become a safe option for doctors seeking information.

## Data Availability

The raw data supporting the conclusions of this article will be made available by the authors, without undue reservation.
