# Supplementary figures and images for: A clinician-based comparative study of large language models in answering medical questions: the case of asthma
Source: Front Pediatr. 2025 Apr 25;13:1461026. doi: 10.3389/fped.2025.1461026 (PMC12062090; doi:10.3389/fped.2025.1461026)

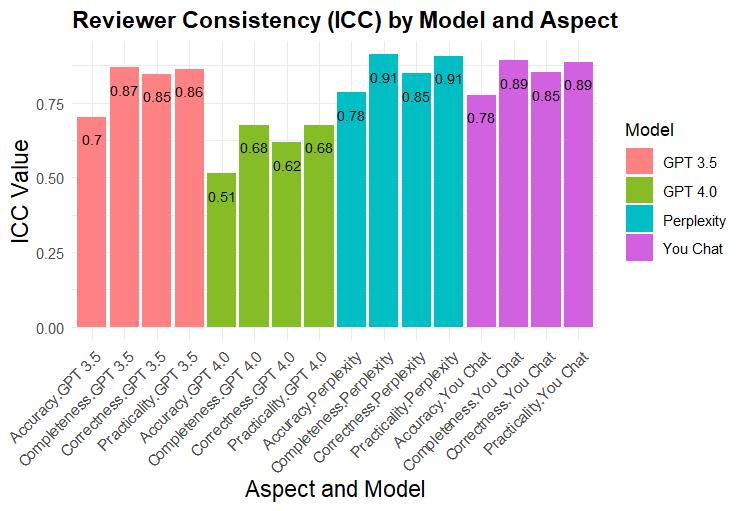

Supplement: Supplementary file 1 [file Image1.tif]
